# Longitudinal trends in prostate cancer incidence, mortality, and survival of patients from two Shanghai city districts: a retrospective population-based cohort study, 2000–2009

**DOI:** 10.1186/1471-2458-14-356

**Published:** 2014-04-14

**Authors:** Yi Hu, Qi Zhao, Jianyu Rao, Haiju Deng, Hong Yuan, Biao Xu

**Affiliations:** 1 Department of Epidemiology, School of Public Health, Key Laboratory of Public Health Safety, Ministry of Education, Fudan University, Shanghai 200032, China; 2 David Geffen School of Medicine and Jonsson Comprehensive Cancer Center, UCLA, Los Angeles, CA, USA; 3 Center for Disease Prevention and Control, Putuo District, Shanghai, China; 4 Center for Disease Prevention and Control, Jiading District, Shanghai, China

**Keywords:** Prostate cancer, Incidence, Mortality, Survival, China

## Abstract

**Background:**

Prostate cancer is the fifth most common cancer affecting men of all ages in China, but robust surveillance data on its occurrence and outcome is lacking. The specific objective of this retrospective study was to analyze the longitudinal trends of prostate cancer incidence, mortality, and survival in Shanghai from 2000 to 2009.

**Methods:**

A retrospective population-based cohort study was performed using data from a central district (Putuo) and a suburban district (Jiading) of Shanghai. Records of all prostate cancer cases reported to the Shanghai Cancer Registry from 2000 to 2009 for the two districts were reviewed. Prostate cancer outcomes were ascertained by matching cases with individual mortality data (up to 2010) from the National Death Register. The Cox proportional hazards model was used to analyze factors associated with prostate cancer survival.

**Results:**

A total of 1022 prostate cancer cases were diagnosed from 2000 to 2009. The average age of patients was 75 years. A rapid increase in incidence occurred during the study period. Compared with the year 2000, 2009 incidence was 3.28 times higher in Putuo and 5.33 times higher in Jiading. Prostate cancer mortality declined from 4.45 per 10^5^ individuals per year in 2000 to 1.94 per 10^5^ in 2009 in Putuo and from 5.45 per 10^5^ to 3.5 per 10^5^ in Jiading during the same period. One-year and 5-year prostate cancer survival rates were 95% and 56% in Putuo, and 88% and 51% in Jiading, respectively. Staging of disease, Karnofsky Performance Scale Index, and selection of chemotherapy were three independent factors influencing the survival of prostate cancer patients.

**Conclusions:**

The prostate cancer incidence increased rapidly from 2000 to 2009, and prostate cancer survival rates decreased in urban and suburban Chinese populations. Early detection and prompt prostate cancer treatment is important for improving health and for increasing survival rates of the Shanghai male population.

## Background

Data from the Global Estimates of Cancer 2008 [1] indicate that the age standardized incidence rate (ASR) of prostate cancer in China was 4.3 per 10^5^. This rate was much lower than the rates in European and American countries [e.g., United States (ASR of 83.8 per 10^5^)] and was also lower compared with other Asian countries [e.g., Singapore (ASR of 20.0 per 10^5^)]. Meanwhile, the current increasing trend compared with 2002 (1.7 per 10^5^) [2] has begun to exceed the rates in some neighboring countries [e.g., India (ASR of 3.4 per 10^5^), Thailand (ASR of 4.2 per 10^5^), and Bangladesh (ASR of 1.7 per 10^5^)]. However, because of the limitations of the cancer registration database, there is very limited detailed information on prostate cancer incidence and survival in the Chinese population. Remarkable social and economic development, population aging, and life style changes have occurred in China [3]. Therefore, it is important to identify the observed increasing trends of prostate cancer incidence and treatment outcome in defined geographic populations.

Shanghai is one of the most developed Chinese cities. Vital statistical data have been available for decades, but the Shanghai Cancer Registry has only been in operation since 1972. There is a well-developed, community-based cancer management system in Shanghai, wherein most cancer patients are monitored by local community health providers. The cancer management system facilitates longitudinal prostate cancer trend analysis of incidence, mortality, and survival.

The objective of this study was to describe longitudinal trends of prostate cancer incidence and mortality, to determine survival status, and to identify factor(s) associated with treatment outcomes. Ten years (2000 to 2009) of Cancer Registry data for two Shanghai districts were used for the analysis.

## Methods

### Study sites and subjects

This retrospective population-based cohort study was carried out in Putuo and Jiading, 2 of the 7 districts in Shanghai. The districts were selected retrospectively based on population characteristics and willingness to participate in the study. Putuo is an industrial area with a resident population of 870,046 individuals in 2009. Jiading is located in a Shanghai suburb and had a population of 546,907 individuals in 2009. The two selected Shanghai areas reflected the composition of the urban and suburban resident populations of the city.

The study subjects were resident prostate cancer patients reported during 2000–2009 in these two districts. Only local Shanghai residents were reported to the Shanghai Cancer Registry. Migrant and temporary residents were not included in the study cohort.

The study protocols were approved by the ethics committee of the School of Public Health, Fudan University (institutional review board-approved protocol number: #04-03-0011).

### Data source

Information on prostate cancer patients was extracted from three databases (i.e., the Shanghai Cancer Registry, the Shanghai Cancer Follow-up database, and the Shanghai Vital Statistics database at the district Center for Disease Prevention and Control (CDC)). Incident cancer cases were reported by Shanghai hospitals to the district CDC and were registered in the Cancer Registry. The identified cases were verified by community health center personnel, and the follow-up data were managed by the CDC. Information in the Cancer Registry database included the patient’s demographic and cancer diagnosis (both clinical and pathologic diagnosis if applicable) data. The cancer-related data included information on disease severity (clinical stage), primary treatment, and active patient follow-up of vital status. Follow-up data included cause of death. To ensure completeness, registry staff validated hospital discharge and death certificate data for registered cases. Prostate cancer death statistics (2000–2010) were extracted from the district CDC Shanghai Vital Statistics database and included age-specific information. The Karnofsky score (KPS) was used to assess quality of life characteristics. The KPS described the ability of each patient to carry out activities on a scale of 0 to 100%. Cancer stage was assessed using the American Urological Association (AUA) prostate cancer staging system [4]. Stages 1–4 were categorized based on primary tumor size relative to prostate size, and involvement of adjacent structures.

### Estimation of incidence and mortality

Considering the differences in population age structure among countries and among different areas in China, age-adjusted incidence and mortality rates of prostate cancer (for all ages) were calculated using the world standard population age distribution (World Health Organization, 2000) and the national standardized population (China Population Census, 2000). Results were presented as the world standardized rate (WSR) per 10^5^ and the national standardized rate (NSR) per 10^5^. The mortality/incidence (M/I) ratio was calculated using the crude and the standardized rates. Relative percentage survival was estimated using $1-\frac{\mathrm{mortality}\left(M\right)}{\mathrm{incidence}\left(I\right)}$. The ratio, $1-\frac{M}{I}$, is typically, but not always, a number between 0 and 100%. A value of 0% indicated extremely poor survival, and a value of 100% indicated excellent survival [5].

### Tests for trends in incidence and mortality

To estimate the longitudinal prostate cancer trends in the two districts, the estimated annual percentage change (EAPC) and 95% confidence intervals (CIs) for incidence and mortality were calculated [6,7]. Using calendar year as a regression variable, a linear regression model was applied to describe the overall changes in trend by fitting the parameter using the equation *y* = m*x* + b, where *y* was ln (rate), and *x* was the calendar year. Based on the assumption that the null hypothesis of the EAPC = 0 was equivalent to the null hypothesis of the slope of the regression line = 0, **EAPC** = **100** × (*e*^*m*^ ‒ **1**) and the 95% CI of **EAPC** = **100** × (*e*^*m* ± *SEm*^ − 1) . The standard error of *m* (SE*m*) was obtained from the fit of the regression line. This calculation assumed that the rates increased or decreased at a constant rate over the entire period. Statistical significance was assessed using the two-tailed test at a given test level.

### Data analysis of survival

Yearly and 5-year survival rates after prostate cancer diagnosis were calculated and stratified by disease severity and age at diagnosis. Survival times were measured from the date of initial diagnosis to the date of death. Yearly survival rates and effects of prognostic covariates were evaluated using the Kaplan–Meier method and the log-rank test within strata defined by age (50–59, 60–69, 70–79, and ≥80), treatment, and KPS (0–50, 50–80, and 80–100) categories. The hazard ratio (HR) and 95% CI were estimated using a multivariate Cox proportional hazard model. A *p*-value < 0.05 was considered to be statistically significant. All tests were performed using SPSS 11.0 (SPSS, Chicago, IL, USA).

## Results

### Demographic and clinical characteristics of prostate cancer patients

A total of 1022 (677 in Putuo and 345 in Jiading) prostate cancer cases aged ≥20 years were identified for the 2000–2009 period, with 43.9% in Putuo and 40.5% in Jiading under 60 years of age. The median age at diagnosis was 75 years (interquartile range: 71–80 years) in Putuo and 75 years (interquartile range:68–80 years) in Jiading (ICD-10 code = C61). For T, N, and M staging, there were 69.8%, 71.2%, and 69.0% missing in Putuo and 72.5%, 19.7%, and 19.1% missing in Jiading, respectively. Overall, 39.7%, 32.8%, 15.2%, and 12.3% of patients had stage T1, T2, T3, and T4 disease in the Putuo district, and 45.3%, 34.7%, 11.6%, and 8.4% of patients had stage T1, T2, T3, and T4 disease in the Jiading district, respectively. Tumor cells were present in the regional or distant lymph nodes in 22.1% of the patients from Putuo and 6.1% of the patients from Jiading (Table 1). Of the 814 patients (539 in Putuo, 275 in Jiading) for whom staging information was available, the distributions of AUA stages 1–4 were 25.8%, 27.5%, 18.0%, and 28.7% in the Putuo patients, and 27.6%, 26.5%, 19.6%, and 26.3% in the Jiading patients, respectively.

**Table 1:** Clinical characteristic of prostate cancer patients during 2000–2009 in Putuo and Jiading

**Variable**	**Putuo (*n*** **= 677)**		**Jiading (*n*** **= 345)**	
	**No.**	**%**	**No.**	**%**
**Vital status at the end of 2010**				
Alive	391	57.8	188	54.5
Dead of all causes	286	42.2	157	45.5
**AUA staging**^**a**^				
1	139	25.8	76	27.6
2	148	27.5	73	26.5
3	97	18.0	54	19.6
4	155	28.7	72	26.3
**TNM staging system**				
**T Stage**^**b**^				
T1	81	39.7	43	45.3
T2	67	32.8	33	34.7
T3	31	15.2	11	11.6
T4	25	12.3	8	8.4
**N stage**^**c**^				
N0	152	77.9	260	93.9
N1	33	16.9	12	4.3
N2	8	4.1	5	1.8
N3	2	1.1	0	0.0
**M stage**^**d**^				
M0	151	71.9	268	96.1
M1	59	28.1	11	3.9
**Treatment by prostatectomy**				
None	141	20.8	17	4.9
Prostatectomy	233	34.4	202	58.6
Palliative treatment	26	3.9	18	5.2
Others	277	40.9	108	31.3

^a^AUA staging information was missing from 138 patients in Putuo and 70 in Jiading.

^b^T staging information was missing from 473 patients in Putuo and 250 Jiading.

^c^N staging information was missing from 482 patients in Putuo and 68 in Jiading.

^d^M staging information was missing from 467 patients in Putuo and 66 in Jiading.

After diagnosis, 34.4% (233/677) of the patients from Putuo and 58.6% (202/345) of the patients from Jiading obtained a prostatectomy, and 141 (20.8%) and 17 (4.9%) of the patients received no cancer-specific treatment. The analysis of the vital statistics data received from the district CDCs revealed that 391 (57.8%) of the patients from Putuo and 188 (54.5%) of the patients from Jiading were alive in December, 2010.

### Trend and point estimation of incidence, mortality, and relative survival

**Figure 1:**
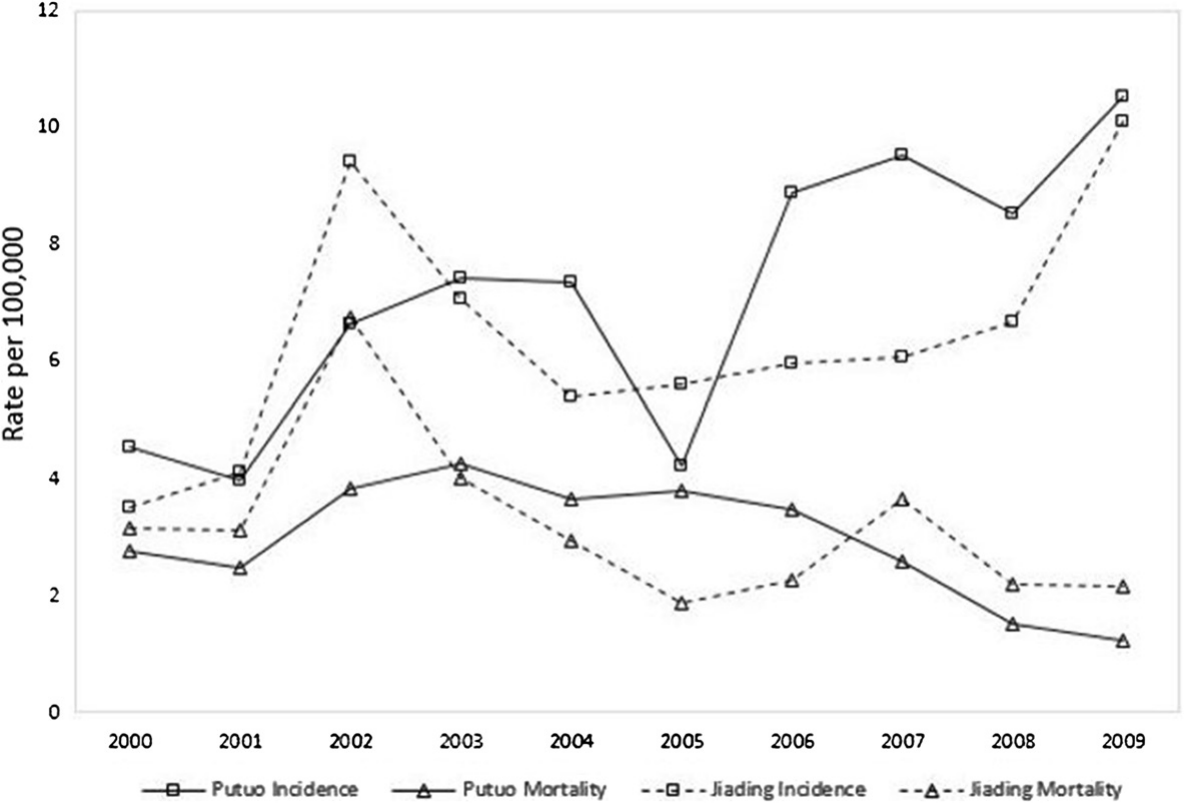
Trend of prostate cancer national standardized rate of incidence and mortality in the male population during 2000–2009.

The results for the analysis of longitudinal trends of prostate cancer incidence indicated that there was a remarkable increase in incidence in Putuo and Jiading (Figure 1). In Putuo, the crude incidence rate (CIR) for prostate cancer increased steadily (EAPC = 13.0%; 95%CI: 9.6–16.5%) from 2000–2009. There was a similar total increase in Jiading (EAPC = 14.1%; 95% CI: 12.4–15.9%). However, the increase in Jiading mainly occurred from 2000–2002 and 2008–2009, and there was little change in CIR from 2003–2008. There were similar trends for NSR and WSR. The EAPCs for NSR and WSR were 7.7% (95% CI: 4.0–11.5%) and 8.5% (95% CI: 5.0–12.2%) in Putuo and 9.1% (95% CI: 7.3–11.0%) and 10.1% (95% CI: 8.2–12.0%) in Jiading, respectively (Table 2).

**Table 2:** Estimated annual percentage change (EAPC) of prostate cancer incidence and mortality during 2000–2009 in Jiading and Putuo

**Rates**	**Putuo district**					**Jiading district**				
	**M**	**SEm**	**EAPC (%)**	***p***	**95% CI (%)**	**M**	**SEm**	**EAPC (%)**	***p***	**95% CI (%)**
Incidence										
Crude	0.122	0.031	13.0	0.005^a^	9.6 ~ 16.5	0.132	0.015	14.1	0.0001^a^	12.4 ~ 15.9
NSR	0.074	0.035	7.7	0.067	4.0 ~ 11.5	0.096	0.017	9.1	0.001^a^	7.3 ~ 11.0
WSR	0.082	0.033	8.5	0.038^a^	5.0 ~ 12.2	0.087	0.017	10.1	0.0001^a^	8.2 ~ 12.0
Mortality										
Crude	−0.402	0.328	−33.1	0.253	−20.1 ~ −45.2	−0.167	0.247	−15.4	0.517	−10.7 ~ −27.4
NSR	−0.272	0.168	−23.8	0.144	−12.7 ~ −41.7	−0.223	0.148	−20.0	0.172	−12.9 ~ −36.7
WSR	−0.498	0.292	−39.2	0.127	−23.7 ~ −45.7	−0.115	0.164	−10.9	0.503	−5.2 ~ −24.8
Relative survival										
Crude	0.060	0.023	6.2	0.031^a^	3.8 ~ 8.7	0.056	0.018	5.8	0.013^a^	3.9 ~ 7.7
NSR	0.063	0.024	6.2	0.031^a^	4.0 ~ 9.1	0.065	0.012	6.7	0.001^a^	5.4 ~ 8.0
WSR	0.060	0.021	6.2	0.02^a^	4.0 ~ 8.4	0.065	0.011	6.7	0.0001^a^	5.6 ~ 7.9

^a^*p* < 0.05.

**Figure 2:**
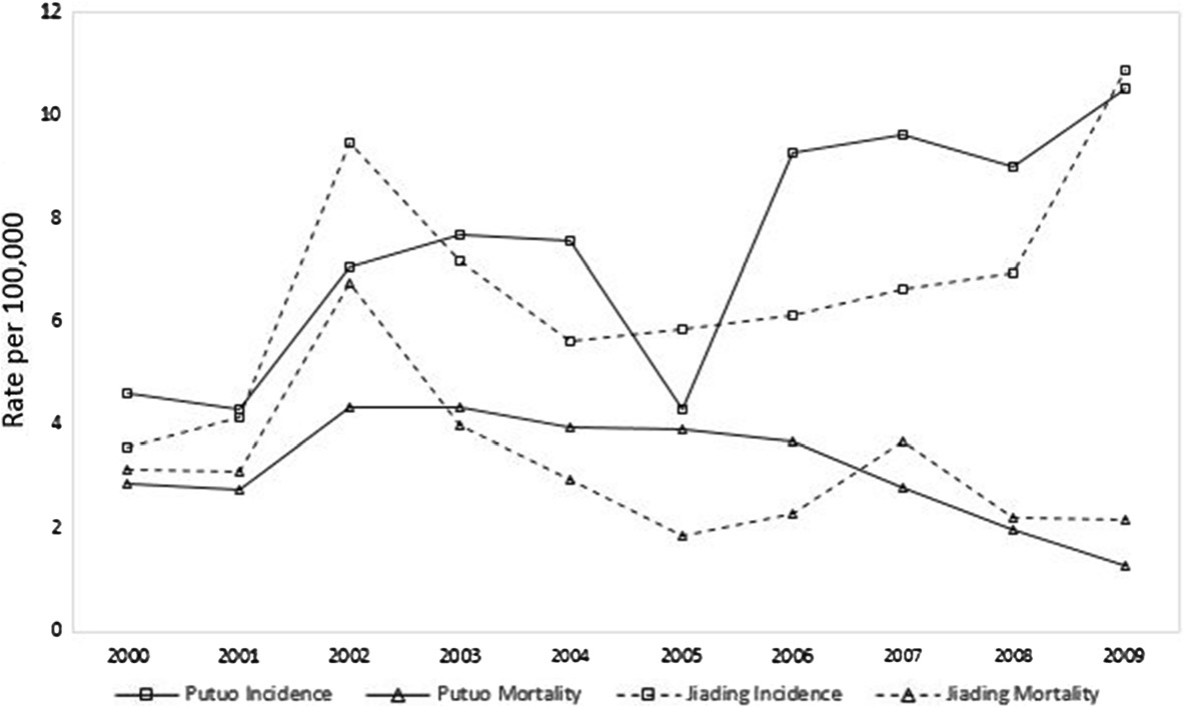
Trend of prostate cancer world standardized rate of incidence and mortality in the male population during 2000–2009.

Prostate cancer mortality rates varied greatly throughout the study period in both of the districts. The highest rate (8.36 per 10^5^) in Putuo occurred in 2005 (Figure 2). In Jiading, the highest mortality rate (11.25 per 10^5^) occurred in 2002. However, there was a general decrease in trend, with an EAPC of −33.1% (95% CI: −20.1 to −45.2%) in Putuo and −15.4% (95% CI: −10.7 to −27.4%) in Jiading. Similar decreasing trends occurred in NSR values (−23.8% in Putuo and −20.0% in Jiading) and WSR (−39.2% in Putuo and −10.9% in Jiading; Table 2).

In general, the relative survival rate increased in both of the districts. The EAPC was 6.2% for the crude (95% CI: 3.8–8.7%), NSR (95% CI: 4.0–9.1%), and WSR (95% CI: 4.0–8.4%) estimates in Putuo and was 5.8% (95% CI: 3.9–7.7%), 6.7% (95% CI: 5.4–8.0%) and 6.7% (95% CI: 5.6–7.9%) for the three estimates, respectively, in Jiading (Table 3).

**Table 3:** Cox Regression analysis on factors influencing survival of prostate cancer patients diagnosed in 2000–2009 in Jiading and Putuo

**Variables**		**Putuo**			**Jiading**	
	**Mean (95% CI)**	**HR (95% CI)**	***p***	**Mean (95% CI)**	**HR (95% CI)**	***p***
**Age at diagnosis (yrs)**						
50-	7.6(5.84-9.28)	1		6.1(4.33-7.92)	1	
60-	6.8(5.87-7.68)	1.4(0.59-3.09)	0.477	5.5(4.50-6.42)	1.6(0.49-5.28)	0.438
70-	6.3(5.84-6.74)	1.5(0.69-3.15)	0.323	6.0(5.25-6.69)	1.6(0.50-5.13)	0.428
80-	4.4(3.88-5.00)	2.4(1.13-5.28)	0.023^a^	3.9(3.10-4.61)	2.5(0.75-8.04)	0.138
**AUA Stage**						
1	6.9(6.05-7.76)	1	1	7.1(5.97-8.23)	1	
2	7.1(6.16-7.96)	1.0(0.55-1.82)	0.987	5.7(4.64-6.69)	1.9(0.70-5.13)	0.209
3	6.6(5.13-8.03)	1.1(0.51-2.36)	0.810	4.0(2.89-5.07)	3.0(1.01-8.94)	0.049^a^
4	3.0(2.45-3.56)	3.9(2.33-6.57)	0.0001^a^	3.8(2.54-5.08)	2.84(1.03-7.81)	0.044^a^
**Curative treatment**						
None	6.4(5.76-7.06)	1		4.1(1.91-6.19)	1	
Prostatectomy	5.8(5.26-6.38)	1.1(0.76-1.51)	0.696	7(6.36-7.62)	0.5(0.19-0.93)	0.026^a^
palliative treatment	7.1(5.49-8.78)	0.7(0.30-1.64)	0.419	6.1(4.77-7.49)	0.6(0.18-1.81)	0.342
Others	5.9(5.33-6.49)	1.3(0.94-1.83)	0.106	2.7(1.97-3.38)	1.6(0.64-4.12)	0.314
**KPS**						
0-	1.3(0.82-1.74)	1		4.6(3.21-5.89)	1	
50-	3.1(2.29-3.96)	0.6(0.33-1.11)	0.430	5.0(3.83-6.19)	0.6(0.26-1.16)	0.118
80-	6.6(6.25-6.96)	0.3(0.17-0.48)	0.001^a^	5.0(4.41-5.60)	0.8(0.47-1.26)	0.299

^a^*p* < 0.05.

**Figure 3:**
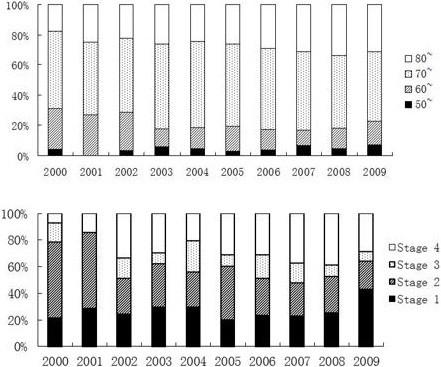
The age and stage stratified distributions of prostate cancer incidence of each year.

Throughout the study period, the 60–70 year age group accounted for the highest proportion of incidence, ranging from 57.0% in 2004 to 46.0% in 2009. A potential increasing trend in incidence occurred in the 50–60 year age group (EAPC = 40.1% (95% CI: 20.0–60.2%), and this trend was statistically significant (*p* = 0.047) (Figure 3).

AUA stage 1 and stage 2 cancers accounted for the majority of the estimated incidence values. The highest level of AUA stage 1 occurred in 2009 (47.1%), and the highest level of AUA stage 2 occurred in 2001 (57.1%). Meanwhile, there was a steady trend in stage 4 cancer for proportion of incidence (EAPC = 8%; 95% CI: 6.9–12.6%, *p* = 0.291).

### Survival analysis

The results for analysis of life tables indicated that the respective overall 1-year and 5-year survival rates were 95% and 56% in Putuo, and 88% and 51% in Jiading. The log-rank analysis revealed that there were significant differences in prostate cancer survival rate between patients from these two districts (*p* = 0.043). The median survival time was 5.16 years (95% CI: 5.02–6.18) in Putuo and 5.06 years (95% CI: 4.94–5.93) in Jiading.

The results of the univariate analysis using log-rank comparisons indicated that cancer stage, age, KPS, and treatment methods were associated with survival. Furthermore, a multivariate analysis using a Cox proportional hazards model revealed that KPS and cancer stage were independent predictive factors for prostate cancer survival (Table 3). Patients from Putuo with a KPS value >80 had a lower HR for death compared with patients with a KPS value of 0–50 (HR, 0.3; 95% CI: 0.17–0.48). Compared with stage 1 patients, stage 3 and stage 4 patients from Jiading (HR_3_ = 3.0, 95% CI: 1.01–8.94; HR_4_ = 2.84, 95% CI: 1.03–7.81) and stage 4 patients from Putuo (HR_4_ = 3.9, 95% CI: 2.33–6.57) had significantly higher HRs for death.

## Discussion

In the 1970s, Shanghai established a cancer registration system that includes the entire resident population. The city-wide cancer registration system documents cancer cases for surveillance, management, and control purposes. The present study used data from this system to determine the longitudinal trends in prostate cancer, including incidence, mortality, and survival status.

The results of this study indicated that there was a trend toward an increased prostate cancer incidence in Shanghai during the past decade. Interpretation of prostate cancer incidence time trends must consider exposure risk factors, population shifts, improvement in case detection, and the impact of PSA screening of asymptomatic patients. The well-established risk factors for prostate cancer are age, race/ethnicity [8], family history [9,10], diet [11,12] and obesity [13,14]. Population demographics are changing significantly in China in association with economic development, especially in urban areas. The proportion of men older than age 65 was 6.71% in Shanghai in 2000 and increased to 16.6% in 2012. Male life expectancy also increased from 78.77 to 79.82 years. Regardless of age adjustment, this trend indicating increased prostate cancer incidence suggests that the number of prostate cancer cases have increased over the past ten years. There have also been lifestyle changes in the relatively rich cities such as Shanghai, which are reflected by an increased obesity burden. Obesity and overweight have become epidemic in urban populations in China [15], and have been proven to be associated with an increased risk of cancer [16], diabetes [17] and hypertension [18]. The increase in prostate cancer incidence in Shanghai might be attributable to the fat-rich diet consumed by the population.

Technology advances and better access to health care are important for the improvement of cancer detection. The increase in prostate cancer incidence might be a result of the application of echo-guided biopsy and PSA testing in symptomatic or asymptomatic patients. There is currently no active PSA screen program in Shanghai, but PSA testing is widely used, especially in older men who present with urological symptoms. As reflected in this study population, most of the patients who receive PSA testing are symptomatic. The availability of universal health care coverage for Shanghai residents also likely has contributed to the healthcare community’s heightened awareness of prostate cancer symptoms. Therefore, changing dietary habits and improved access to health care (including PSA testing) may have contributed to the increased prostate cancer incidence observed in this study. However, the exact contribution of each factor is difficult to quantify.

A relative reduction in mortality from prostate cancer was a finding from our study. This reduction could be explained by improvements in diagnosis and/or early detection, and the subsequent use of radical prostatectomy [19,20]. The results of our study indicated that there was a trend towards early prostate cancer detection. The proportion of stage 1 cancer was 42% in 2009 and was 20% in 2001. High 5-year survival rates have been reported for localized prostate cancers (stage 1 or 2) [21,22], most likely because there are a number of effective treatment options (e.g., radical prostatectomy, radiation). Mortality from high stage disease increases substantially when therapeutic options are rather limited. The results of Scandinavian Prostate Cancer Group Study Number 4 [22] indicated that there was an absolute reduction in mortality of 6 percentage points among men who underwent radical prostatectomy. Similarly, the results of our study suggest that radical prostatectomy reduced prostate cancer mortality. Taken together, these observations indicate that early detection and proper treatment could reduce the prostate cancer mortality. However, over-diagnosis and subsequent treatment may result in side effects (e.g., incontinence and impotence), and adversely affect quality of life.

It was striking to find that compared with the industrial countries that have high rates of survival from prostate cancer, the 1-year and 5-year survival rates were only 95% and 56% in Putuo, and 88% and 51% in Jiading, respectively. The median survival time was approximately 5 years in both districts. Findings from this analysis also suggest that an initial treatment using prostatectomy and/or chemotherapy may potentially be associated with prolonged survival. Surgery might be effective for the prevention of the spread of prostate cancer. A history of chemotherapy was a predictor that was associated with improved survival in patients undergoing surgery, compared with patients without any treatment. The survival benefit from chemotherapy could be due to tumor cell destruction during prior chemotherapy. Baseline differences in KPS and staging did have an effect on survival. Prostate cancer patients with KPS ≥ 80 and tumors graded at below a stage 3 were more likely to survive. These characteristics are consistent with less pathologically aggressive disease and a smaller tumor mass [23]. These findings suggest that more efforts should be aimed at early detection of symptomatic patients, and that treatment effects should be verified systematically. Progression in disease pathology should be well documented, and appropriate treatment follow-up is critical.

There has recently been much debate on the need for prostate cancer intervention programs in industrialized countries because it seemed as if early detection and cure were no longer problems in these countries. Almost one-half of the patients included in this study died within five years after diagnosis, which indicates that the value of early detection intervention deserves further discussion. However, most of the patients were older men, so over-diagnosis is a greater concern because of their limited life expectancy. This observation and the findings of the stable trend in incidence in the younger group and the increasing trend in relative survival suggest that a prostate screening program might have limited effects.

The results of this study also revealed that there was an increasing trend in cancer stages 1 and 2. Surgery and chemotherapy do improve survival among prostate cancer patients, and it is likely that treatment with the intention to cure has contributed to the reductions in mortality. Educational programs that instill adequate awareness of early symptoms of prostate cancer in special high risk populations (e.g., males >70 years of age) should be developed and implemented. Early prostate cancer detection should improve when the urban population is well-formed.

There were some limitations of this study. Especially for the subjects enrolled in 2009, follow-up time might have not been sufficient for adequate evaluation of outcomes. Therefore, mortality might have been slightly underestimated. The pathological diagnosis (TNM staging) was missing from the records for a relatively high proportion of subjects. Gleason score data were also not included in the registry. Therefore, we were unable to obtain a comprehensive understanding of prostate cancer pathology. Management of the Cancer Registry and Cancer Follow-up databases should be changed so that the quality of the system improves.

## Conclusions

The world population adjusted incidence of prostate cancer was relatively low in the Putuo and Jiading districts of Shanghai in 2009. However, an increase in prostate cancer incidence occurred between 2000 and 2009, which included an estimated annual proportional change of approximately 13.0% in Putuo and 14.1% in Jiading. The prostate cancer survival rate was is disappointedly low; the 5-year survival rate was 56% and 51% for Putuo and Jiading districts, respectively. Health education on early detection and prompt treatment of prostate cancer is of great importance for improving health and prolonging life of the adult male population in Shanghai.

## Competing interests

The authors declare that they have no competing interests.

## Authors’ contributions

BX, YH and QZ developed the study design. YH, QZ, JR, HD, HY organized the data and conducted the statistical analyses. YH and BX were the primary writers of the manuscript and all authors were involved in editing the manuscript. All authors read and approved the final manuscript.

## Pre-publication history

The pre-publication history for this paper can be accessed here:

http://www.biomedcentral.com/1471-2458/14/356/prepub
